# Quantitative Analysis and Molecular Docking Simulation of Flavonols from *Eruca sativa* Mill. and Their Effect on Skin Barrier Function

**DOI:** 10.3390/cimb46010025

**Published:** 2024-01-02

**Authors:** Jihye Park, Wonchul Choi, Jayoung Kim, Hye Won Kim, Jee-Young Lee, Jongsung Lee, Bora Kim

**Affiliations:** 1 Department of Chemistry, The Graduate School, Mokwon University, Daejeon 35349, Republic of Korea; rmarkd545@naver.com (J.P.); kjo08@naver.com (J.K.); kxxhxxwxx98@naver.com (H.W.K.); 2 Interdisciplinary Program in Biocosmetics, Sungkyunkwan University, Suwon 16419, Republic of Korea; wonchulc@g.skku.edu; 3 Structure Based Drug Design Laboratory, New Drug Development Center, Daegu Gyeongbuk Medical Innovation Foundation (K-Medi Hub), Daegu 41061, Republic of Korea; jyoung@kmedihub.re.kr; 4 Department of Cosmetics Engineering, Mokwon University, Daejeon 35349, Republic of Korea

**Keywords:** *Eruca sativa*, skin barrier function, peroxisome proliferator-activated receptor-α, anti-inflammation, docking simulation

## Abstract

*Eruca sativa* is a commonly used edible plant in Italian cuisine. *E. sativa* 70% ethanol extract (ES) was fractionated with five organic solvents, including n-hexane (EHex), chloroform (ECHCl_3_), ethyl acetate (EEA), n-butyl alcohol (EBuOH), and water (EDW). Ethyl acetate fraction (EEA) had the highest antioxidant activity, which was correlated with the total polyphenol and flavonoid content. ES and EEA acted as PPAR-α ligands by PPAR-α competitive binding assay. EEA significantly increased cornified envelope formation as a keratinocyte terminal differentiation marker in HaCaT cells. Further, it significantly reduced nitric oxide and pro-inflammatory cytokines (IL-6 and TNF-α) in lipopolysaccharide-stimulated RAW 264.7 cells. The main flavonol forms detected in high amounts from EEA are mono-and di-glycoside of each aglycone. The main flavonol form of EEA is the mono-glycoside of each aglycone detected, and the most abundant flavonol mono-glycoside is kaempferol 3-glucoside 7.4%, followed by quercetin-3-glucoside 2.3% and isorhamnetin 3-glucoside 1.4%. Flavonol mono-glycosides were shown to be a potent PPAR-α ligand using molecular docking simulation and showed the inhibition of nitric oxide. These results suggest that the flavonol composition of *E. sativa* is suitable for use in improving skin barrier function and inflammation in skin disorders, such as atopic dermatitis.

## 1. Introduction

The skin is the outermost organ that protects against ultraviolet radiation, environmental pollutants, harmful microbes, and dry environments. Most of the protective function is provided by the epidermis, with various layers, including the stratum corneum (SC), which consists of two major structural components, corneocytes and intercorneocyte lipids, stratum granulosum, stratum spinosum, and stratum basale. The epidermis is a stratified squamous epithelium that acts as a barrier against chemical, physical, and biological agents [1]. The SC of terminally differentiated cornified cells is the outermost epidermis, which forms the barrier properties of the skin [2]. Atopic dermatitis (AD), a skin barrier disease, is a chronic inflammatory skin disorder characterized by eczematous and pruritic skin. Enormous changes in the composition and function of the epidermal barrier influence the initiation and maintenance of skin inflammation in AD [3].

Peroxisome proliferator-activated receptors (PPARs) are ligand-activated transcription factors belonging to the nuclear receptor family such as PPAR-α, PPAR-β/δ, and PPAR-γ. PPAR-α is highly expressed in many tissues, including the heart, kidney, liver, skeletal muscle, and epidermis, where it is an important regulator of lipid metabolism [4]. Topical treatment with PPAR-α agonists was found to restore epidermal homeostasis in skin barrier disruption models. Thus, PPAR-α agonists have been extensively studied in keratinocyte differentiation and the epidermal permeability barrier, and it has been shown that topical treatment with PPAR ligands promotes differentiation in the murine epidermis [5]. The PPAR-α agonists, WY14643 and ciglitazone, elevated the expression of the cornified envelope (CE)-associated proteins such as involucrin, transglutamase-1, and others [6]. The CE completes the epidermal permeability barrier during the terminal differentiation of keratinocytes. Therefore, a search for new agonists is required to determine whether activators of PPAR-α can activate the rate of keratinocyte differentiation. Inflammation is a complex process regulated by the activation of various immune cells. In particular, macrophages play a key role in mediating many different inflammation phenomena, including the expression of pro-inflammatory cytokines and inflammatory mediators such as tumor necrosis factor-α (TNF-α), interleukin-1β (IL-1β), nitric oxide, prostaglandinE_2_, and regulation on activation. It has been reported that several components affected the inflammatory phenotype of macrophages by activating PPARs [4]. Therefore, screening for new agonists is required to determine whether activators of PPAR-α can improve keratinocyte differentiation. Topical treatment with a PPAR-α activator showed potent anti-inflammatory effects in an AD model, which was associated with the alleviation of the pro-inflammatory cytokines via TNF-α and IL-6 [7,8]. Therefore, PPAR-α activation and anti-inflammatory activities can improve skin barrier function. 

*Eruca sativa* Mill. is an edible annual plant that inhabits the Mediterranean coast, is referred to as rocket or arugula, and is commonly used in Italian foods [9]. *E. sativa* is known to have anti-inflammatory activity by inhibiting the skin barrier improvement effect and inflammatory cytokines. Further, it contains various phytochemicals, thus its antioxidant, anti-microbial, and PPAR-α agonistic activities [10,11,12,13]. We previously reported that *E. sativa* crude extract has various functions in improving the skin barrier [8]. In this study, we aimed to determine the skin barrier improvement and anti-inflammatory effect of each organic solvent fraction of *E. sativa* and examine whether the major flavonols identified by instrumental analysis and in silico molecular docking simulation are PPAR-α ligands.

## 2. Materials and Methods

### 2.1. Chemicals and Reagents

Sigma-Aldrich (St. Louis, MO, USA) provided 2,2-diphenyl-1-picrylhydrazyl (DPPH), 2′,7′-dichlorofluorescein diacetate (DCF-DA), lipopolysaccharide (LPS), Folin–Ciocalteu (F–C) reagent, gallic acid, AlCl_3_, Griess reagent, NG-Methyl-L-arginine acetate salt (L-NMMA), WY14643, CaCl_2_, and HPLC grade standards of quercetin, kaempferol, isorhamnetin, kaempferol 3-glucoside, isorhamnetin 3-glucoside, quercetin 3-glucoside, and quercetin 3,4′-diglucoside. Solvents for extraction and fractionation (ethanol, n-hexane, chloroform, ethyl acetate, and n-butanol) and formic acid were purchased from Daejung Chemicals & Metals (Siheung, Republic of Korea). Solvents for LC/MS (methanol, acetonitrile) were purchased from Honeywell Burdick & Jackson (Muskegon, MI, USA).

### 2.2. Extraction and Fractionation of E. sativa

*E. sativa* leaves were purchased from the Garak agricultural and marine product wholesale market (Garak-dong, Seoul, Republic of Korea). After washing with purified water, they were chopped and dried for 24 h at 50 °C. Next, the leaves (800 g) were refluxed twice and extracted with 5 L of 70% ethanol for 24 h at 55 °C. The *E. sativa* extract was then filtered through a No. 6 filter paper and vacuum evaporated (Advantec, Tokyo, Japan). Then, 70% ethanolic extract suspended in water was successively extracted with solvents of increasing polarity. The crude ethanolic extract of *E. sativa* (230 g) was fractionated by the solvent partitioning method (800 mL of each step, three times) using its aqueous suspension with the increase of the polarity of the solvent from hexane to n-butyl alcohol. Each fraction was: n-hexane fraction (EHex, 9.6 g); chloroform fraction (ECHCl_3_, 0.4 g); ethyl acetate fraction (EEA, 4.5 g); n-butyl alcohol fraction (EBuOH, 9.6 g); water fraction (EDW, 209.5 g).

### 2.3. Antioxidant Activity of 2,2-Diphenyl-1-Picrylhydrazyl

A 0.2 mM DPPH solution was used to determine the antioxidant capacity of the extracts, and the reaction was carried out for 30 min in an incubator maintained at 25 °C. Lower SC_50_ values (the concentration of each sample for scavenging 50% of radicals) indicated a higher radical scavenging potential. L-ascorbic acid was used as the positive control. 

### 2.4. Total Polyphenol and Flavonoid Content Assay

The total polyphenol content of the extract and its fractions was determined using the Folin–Ciocalteu (F–C) reagent. The samples were mixed with an F–C reagent and 700 mM sodium carbonate. After the mixture had reacted for 30 min in an incubator maintained at 25 °C, absorbance was measured at 765 nm using a microplate reader. The total polyphenol content was expressed in gallic acid equivalent (GAE) mg/g of the extract and each fraction. The total flavonoid content of the extract and each fraction was determined using a modified aluminum chloride colorimetric method. 5% NaNO_2_ was added to the sample and reacted, followed by mixing with 10% AlCl_3_. Lastly, the reaction was performed with 1 N NaOH at 25 °C, and the absorbance was measured at 510 nm using a microplate reader. The total flavonoid content was expressed as mg of quercetin equivalent (QE)/g of extract and each fraction.

### 2.5. Cell Culture 

Cell lines were purchased from the Korea Cell Line Bank (KCLB, Seoul, Republic of Korea). RAW 264.7 and HaCaT cells were cultured in Dulbecco’s modified Eagle’s medium (DMEM, Welgene, Republic of Korea) containing 10% fetal bovine serum (FBS, Gibco, Waltham, MA, USA) and 1% penicillin-streptomycin (Gibco, Waltham, MA, USA) in a humidified atmosphere of 5% CO_2_ at 37 °C. Cytotoxicity was determined using a water-soluble tetrazolium salt-1 (WST-1) assay (Dogenbio, Seoul, Republic of Korea). 

### 2.6. Cornified Envelope Formation

HaCaT cells were seeded at a density of 5 × 10^5^ cells in 60-mm dishes. Cells kept in a medium containing 0.09 mM Ca^2+^ (low calcium, LC) were considered undifferentiated. Cells kept in a medium containing 3.5 mM Ca^2+^ (high calcium, HC) were used as a positive control to represent the differentiated status. After incubation for 24 h, the samples and 3.5 mM CaCl_2_ as a differentiation-inducing control were combined for 5 days. Cell lysate was heated to 95 °C for 15 min, centrifuged at 20,000 rpm for 60 min, and then analyzed at 430 nm. Total protein was determined using the Bradford assay.

### 2.7. PPAR-α Binding Assay

LanthascreenTM TR-FRET-based competitive binding assay kits (Invitrogen, Waltham, MA, USA) were used to evaluate the PPAR-α binding activity of ligands according to the manufacturer’s instructions. The kit uses a terbium-labeled anti-GST antibody, a fluorescent small molecule pan-PPAR ligand (Fluormone™ Pan-PPAR Green, also referred to as tracer), and a human PPAR-α ligand-binding domain that is tagged with glutathione S-transferase (GST) in a homogenous mix-and-read assay format. All assay measurements were performed using an Infinite 200 (Tecan, Mannedorf, Switzerland). WY14643 was used as the positive control.

### 2.8. Docking Study

A possible binding model between PPAR-⍺ and flavonols was predicted using docking simulations based on the complex structures of PPAR-α and its activator. The possible binding of three flavonols (kaempferol 3-glucoside, quercetin 3-glucoside, and isorhamnetin 3-glucoside) was defined, and the known interactions between PPAR-⍺ and the activator were considered. The binding sites of flavonols were expected to be similar to that of known PPAR-⍺ ligands, and two recently reported PDB structures (7E5I.pdb and 7E5H.pdb) were used as references for the docking studies. The initial binding model was determined by a rigid docking study, and molecular mechanics energies combined with the generalized born and surface area (MM-GBSA) were used for the final binding model prediction. The binding free energy is an important parameter of MM-GBSA and is used to determine the binding affinity of drugs to target proteins. MAESTRO 11.8 package (Schrödinger LLC, New York, NY, USA) was used for all calculations using the Glide and Prime modules in a Linux environment with default parameters [14].

### 2.9. Measurement of Intracellular Reactive Oxygen Species

Intracellular reactive oxygen species (ROS) were determined using 2′,7′-dichlorofluorescein diacetate (DCF-DA). RAW 264.7 cells were treated in the presence of 2 μg/mL lipopolysaccharide (LPS) together with samples at 37 °C for 1 h at a density of 2.0 × 10^5^ cell/confocal dish. The increase of fluorescence signal was attributed to a self-oxidization process of DCF-DA, which transferred dichlorodihydrofluorescein (DCFH) to 2′,7′-dichlorofluorescein (DCF) with the presence of DCF. The fluorescent probe, DCF-DA, was used to monitor the intracellular generation of LPS-induced ROS. After 30 min of LPS treatment, DCF-DA 10 μM was added to the cells, which were subsequently incubated for 30 min at 37 °C. The intracellular ROS concentration was monitored using an inverted fluorescence microscope (Zeiss HBO 100, Thornwood, NY, USA).

### 2.10. Measurement of Nitric Oxide, IL-6 and TNF-α Production

Nitric oxide (NO) production in the medium was determined by reacting the sample with the Griess reagent. After RAW 264.7 cells were seeded in a 96-well plate at a concentration of 2.0 × 10^5^ cells/well and incubated for 24 h in a 5% CO_2_ atmosphere and 37 °C, they were stimulated with various concentrations of samples and 2 μg/mL LPS for 24 h. L-NMMA, a NO synthase inhibitor, was used as a positive control. IL-6 and TNF-α were determined using an ELISA kit according to the manufacturer’s instructions (R&D systems, Minneapolis, MN, USA).

### 2.11. Reverse Transcription-Polymerase Chain Reaction (RT-PCR)

Total RNA was isolated from RAW 264.7 cells using the RNeasy Mini Kit (Qiagen, Venlo, The Netherlands). cDNA was synthesized by RT-PCR using the OneStep RT-PCR Kit (Qiagen, Venlo, The Netherlands). The mRNA levels of iNOS and β-actin (housekeeping gene) were measured by PCR with primers (Bioneer, Daejeon, Republic of Korea). The PCR conditions for each gene were as follows: mouse β-actin, 5′-TGGAATCCTGTGGCATCCATGAAAC-3′ and 5′-TAAAACGCAGCTCAGTAACAGTCCG-3′; mouse iNOS, 5′-AGCCCAACAATACAAGATGACCCT-3′ and 5′-TTCCTGTTGTTTCTATTTCCTTTGT-3′. The PCR product sizes for iNOS and β-actin are 407 bp and 350 bp, respectively. Amplification was performed for 25 cycles. Each cycle consisted of denaturation for 1 min at 95 °C, annealing for 1 min at the appropriate primer-specific temperature, and extension for 1 min at 72 °C. An additional incubation at 72 °C for 10 min was executed after the last cycle. The PCR products were subject to electrophoresis on 1.8% agarose gels.

### 2.12. Analysis of Flavonols

Each fraction of the *E. sativa* was analyzed by HPLC-UV-MS. Approximately 0.05 g of each fraction was weighed and ultrasonically extracted with 25 mL of methanol for 30 min. Then, the extract was filtered through a 0.45 μm membrane filter prior to analysis. The identification of flavonols was conducted on the HPLC-UV-MS system (Ultimate3000 UPLC system with PDA detector and LTQ-XL ion trap mass spectrometer, Thermo-Fisher Scientific, Waltham, MA, USA). The chromatographic separation was performed on the analytical column (Hypersil GOLD, 2.1 × 200 mm, 1.9 µm) maintained at 35 °C and by the gradient elution of the mobile phase consisting of 100 mM formic acid in deionized water (mobile phase A) and acetonitrile (mobile phase B) at the flow rate of 0.2 mL/min. The gradient program was from 0 (15% B) to 45 min (40% B), from 75 to 84 min (98% B), and from 85 to 90 min (15% B). Mass spectrometry with an electrospray ionization (ESI) source was conducted in the negative ion mode. The analysis was performed using full scan mode and data-dependent MS2 in the mass range of *m*/*z* 50–1000. The MS settings were as follows: spray voltage, 5.0 kV; sheath gas, 30 Arb; auxiliary gas, 7 Arb; capillary temperature, 275 °C; collision energy of MS2, 35%. The data were gathered using Xcaliver and Chromeleon software version 7.3.2 (Thermo-Fisher Scientific). Quantitative analysis was done by UV detection at 371 nm using appropriate standard reagents.

### 2.13. Statistical Analysis

All data are presented as mean ± standard deviation (SD). Statistical analyses were performed using GraphPad Prism 5.0 (GraphPad Software, San Diego, CA, USA). Comparisons between multiple groups were performed using a one-way analysis of variance with Bonferroni’s comparison of all pairs of columns. Statistical significance was set at *p* < 0.05.

## 3. Results and Discussion

### 3.1. Antioxidant Activity

**Figure 1:**
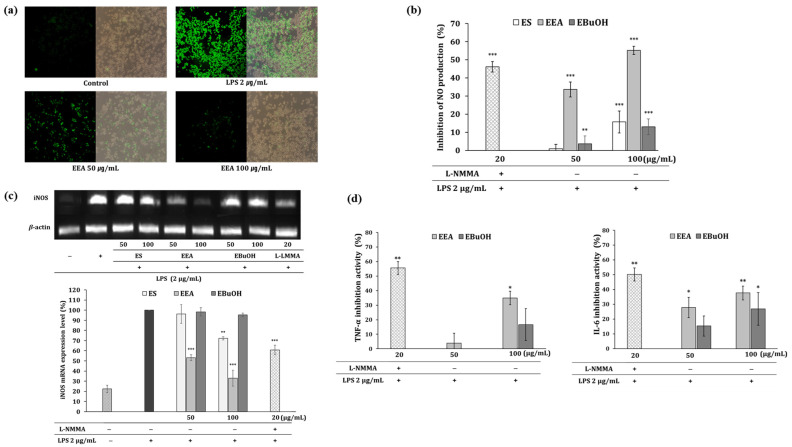
(**a**) The intracellular ROS scavenging activity. Fluorescence images (×200 magnification) of ROS produced by treatment with LPS in RAW 264.7 cells are shown. Dichlorofluorescein (DCF) fluorescence intensity, indicative of LPS-induced oxidant stress, was reduced by EEA 50 and 100 μg/mL. (**b**) Inhibition of NO production. (**c**) Inhibition of iNOS mRNA expression by EEA and EBuOH (**d**) Effect of EEA and EBuOH on inflammatory cytokines, TNF-α, IL-6 by ELISA. Values are presented as the mean ± SD of triplicate measurements (* *p* < 0.05, ** *p* < 0.01, *** *p* < 0.001, compared with negative control).

Antioxidant activity was investigated through the DPPH radical scavenging and ROS detection assays using H_2_DCFDA in LPS-stimulated RAW 264.7 macrophages. EEA and EBuOH showed the highest radical scavenging activities compared to the other fractions. In the DPPH antioxidant assay, the SC_50_ was evaluated at five concentrations (62.5, 125, 250, 500, and 1000 μg/mL). The DPPH scavenging activities of the fractions in decreasing order were as follows: EEA > EBuOH > ES > ECHCl_3_ > EDW > EHEX (Table 1). The intracellular ROS scavenging activity, which was monitored by dichlorofluorescein (DCF) fluorescence intensity, was decreased by LPS-induced oxidative stress in EEA. The green fluorescence intensity was proportional to the ROS levels within the cell cytosol. EEA 50, 100 μg/mL showed a dose-dependent antioxidant effect (Figure 1a). 

### 3.2. Determination of Total Phenolic and Flavonoid Content

Phenolic compounds are synthesized by plants in response to various stresses such as infection, injury, and UV radiation [15]. Flavonoids belong to the polyphenol subgroup with various health-improving effects [16]. Quantification of total phenolic content (TPC) and total flavonoid content (TFC) using calibration curves of gallic acid and quercetin, respectively, showed the greatest abundance in EEA (104.99 ± 5.88 mg GAE/g extract, 74.24 ± 1.67 mg QE/g extract) followed by EBuOH (88.42 ± 2.17 mg GAE/g extract, 53.41 ± 0.33 mg QE/g extract) as summarized in Table 1. These results indicate that EEA and EBuOH contained high flavonoid contents compared to TPC. As such, the most antioxidative fraction was highly correlated with total polyphenol and flavonoid contents.

### 3.3. Anti-Inflammatory Effects

The activation of macrophages by the bacterial surface molecule LPS leads to the production of ROS and NO radicals, which play important roles in inflammation [17]. ES, EEA, and EBuOH showed cell viability of more than 80% in RAW 264.7 cells at concentrations ˂ 100 μg/mL. Treatment of the cells with 100 μg/mL of ES, EEA, and EBuOH significantly inhibited NO production, which is a well-known inflammatory mediator in LPS-induced RAW 264.7 cells (Figure 1b). Treatment with 100 μg/mL EEA significantly also reduced iNOS mRNA expression (Figure 1c). In addition, 100 μg/mL EEA and EBuOH significantly inhibited pro-inflammatory cytokines production such as IL-6 and TNF-α (Figure 1d). Three major flavonol aglycones and their mono glucosides (quercetin, kaempferol, isorhamnetin, quercetin 3-glucoside, kaempferol 3-glucoside, and isorhamnetin 3-glucoside) which were confirmed to inhibit NO production (Table 2) and quantified in EEA (Table 3). Quercetin 3-glucoside showed the strongest anti-inflammatory effect (IC_50_ = 1.81 μM). The anti-inflammatory effect of this fraction was postulated to be a complex synergistic effect of three major flavonol aglycones and their mono glucosides, as the anti-inflammatory effect was better than that of other fractions [18].

### 3.4. CE Formation and PPAR-α Binding Assay

**Figure 2:**
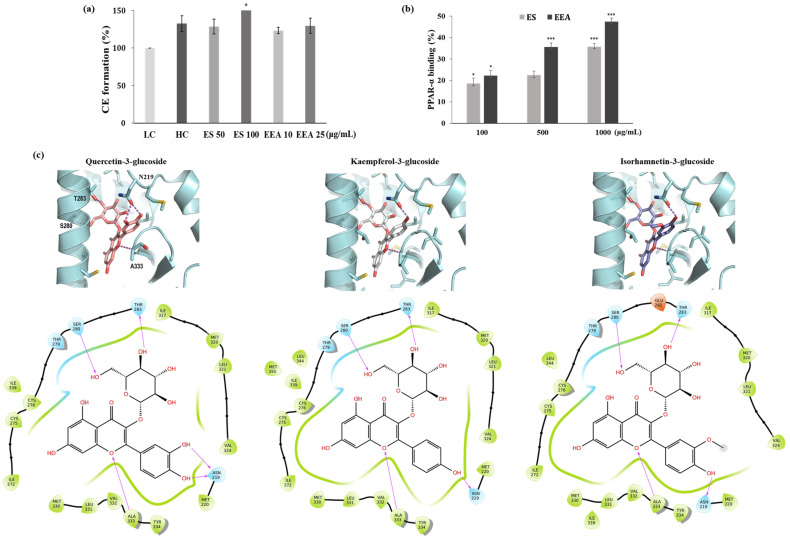
(**a**) Effect of ES and EEA on cornified envelope formation. ES and EEA were co-treated in HaCaT cells under low Ca^2+^ (LC, 0.09 mM) conditions, and high Ca^2+^ (HC, 3.5 mM) treatment was compared as a positive control. Cornified envelope formation composed of insoluble proteins in the cell lysate. (**b**) PPAR-α binding activity by TR-FRET PPAR-α competitive binding assay (**c**) Analysis of interactions between PPAR-α and three flavonol mono-glycoside (Quercetin-3-glucoside, Kaempferol-3-glucoside, Isorhamnetin-3-glucoside). Ligand interaction diagram supported by docking results. Values are presented as the mean ± SD of triplicate measurements (* *p* < 0.05, *** *p* < 0.001, compared with negative control).

To investigate the effects of EEA and ES on keratinocyte differentiation, we measured CE formation as a terminal differentiation marker in HaCaT cells at the low calcium concentration (0.09 mM, LC) [19,20]. When cells were treated with ES and EEA, the CE formation assay was performed at a concentration that resulted in more than 80% cell viability. Compared with the LC, the groups treated with 100 μg/mL of ES significantly showed high CE formation (*p* < 0.05). CE formation was increased by approximately 20–30% following ES and EEA treatments compared to low calcium treatment (Figure 2a). PPAR-α is a ligand-activated transcription factor that plays an important role in epidermal homeostasis. In a nuclear receptor binding assay based on TR-FRET, EEA and ES competitively replaced the binding of labeled PPAR-α ligands by approximately 18–47% (Figure 2b, *p* < 0.001). The positive control, WY14643, showed a significant increase from 10 to 1000 μM. ES and EEA may play a role in activating PPAR-α and promoting keratinocyte differentiation for skin barrier recovery [21].

### 3.5. Binding Model Prediction between PPAR-α and Flavonols

In this study, the initial binding model was determined by rigid docking, and the MM-GBSA calculation, which induces structural changes in the protein according to the binding of the ligand, was used to determine the final binding structure. MM-GBSA is widely used to estimate the binding free energy of small molecules and proteins [22,23]. It is well known that PPAR activators bind to the ligand-binding domain (LBD) of PPARs, and many PPAR-activator complex structures are elucidated by X-ray crystallography. Quercetin-3-glucoside, a possible activator of PPAR-⍺, is capable of binding to PPAR-⍺ LBD with low binding energy. The important key interactions of quercetin-3-glucoside are five hydrogen bonds (H-bonds) with PPAR-⍺ residues. The two hydroxyl moieties on the sugar ring formed H-bonds with the side chains of S280 and T283. The other two hydroxyl groups of the B-ring also participated in a H-bond interaction with N219. Further, an oxygen atom on the A-ring formed an H-bond with the NH backbone of the A333 residue. H-bond interactions are important in determining whether a ligand binds to a target protein [24]. Five H-bonding interactions between PPAR-⍺ and quercetin-3-glucoside played a role in stabilizing the interaction and increasing binding affinity (Figure 2c). Kaempferol-3-glucoside and isorhamnetin-3-glucoside are also well-fitted to activator binding sites on PPAR-⍺ and the binding model is like that of quercetin-3-glucoside. Quercetin-3-glucoside has two hydroxyls on the 3′- and 4-positions of the B-ring, but the rest of the flavonols only have one hydroxyl moiety at the 4′-positon. The absence of hydroxyl moiety on the 3′-position of the B-ring reduced the number of H-bonding interactions than quercetin-3-glucoside. This leads to a diminishment of the binding affinity. The binding free energies resulting from the MM-GBSA calculation support that quercetin-3-glucoside has a slightly higher affinity than the other two flavonols. These results suggest that quercetin-3-glucoside can more strongly bind to PPAR-⍺ and may act as an activator of PPAR-⍺.

### 3.6. Analysis of Flavonols

The identification of the major flavonols of each fraction was performed on HPLC-UV-MS. Based on the retention times, molecular formula, MS2 data, reference standards and previous study [25], 9 major compounds were identified, which include quercetin, kaempferol, isorhamnetin, and glucose derivatives of these three aglycones, as shown in Table 3. The flavonol content of the crude ethanolic extract of *E. sativa* and solvent partitioning fractions were quantitated, as shown in Table 4. The flavonol composition of the solvent fractions of *E. sativa* was determined for the first time. Three main flavonol aglycones such as kaempferol, quercetin, and isorhamnetin, were detected in EEA with comparatively small amounts, which is consistent with our previous results [8]. The main flavonol form of EEA is the mono-glycoside of each aglycone detected, and the most abundant flavonol mono-glycoside is kaempferol 3-glucoside at 7.4%, followed by quercetin-3-glucoside at 2.3%, and isorhamnetin 3-glucoside at 1.4%. The second abundant flavonol form of EEA is the di-glycoside of each aglycone detected, such as kaempferol 3,4′-diglucoside, quercetin 3,4′-diglucoside, and isorhamnetin 3,4′-diglucoside. On the other hand, the most abundant flavonols formed in EBuOH are di-glycosides of the three aglycones, different from those of EEA. Kaempferol 3,4′-diglucoside is the most abundant flavonol in the EBuOH fraction because of the higher polarity of n-butanol than that of ethyl acetate. Flavonol di-glycosides are the main components in EBuOH, EDW, and crude 70% ethanol extract, which is consistent with the studies in the past [26,27,28]. Total flavonol content is comparatively large in EEA (13.2%) and EBuOH (7.7%), which is positively related to the results of the total phenolic and flavonoid content, as shown in Table 1. These results also make it possible to study the correlation between chemicals and biological activity. Furthermore, the biological activity of flavonols can be influenced by their steric hindrances at the active site, resulting in the better biological activity of the EEA fraction enriched with flavonol mono-glycosides than that of other fractions enriched with flavonol di-glycosides.

## 4. Conclusions

Ethyl acetate fraction of *E. sativa* was analyzed to contain high amounts of three flavonols, including kaempferol, quercetin, and isorhamnetin, and their mono-glycosides, which showed the best antioxidant, skin barrier function improvement via PPAR-α activation, and anti-inflammatory effects. The main flavonol form of ethyl acetate fraction is the mono-glycoside of each aglycone detected, and the most abundant flavonol mono-glycoside is kaempferol 3-glucoside at 7.4%, followed by quercetin-3-glucoside at 2.3% and isorhamnetin 3-glucoside at 1.4%. The second abundant flavonol form of ethyl acetate fraction is the di-glycoside of each aglycone detected, such as kaempferol 3,4′-diglucoside, quercetin 3,4′-diglucoside, and isorhamnetin 3,4′-diglucoside. The anti-inflammatory effects of ethyl acetate and n-butyl alcohol fractions were postulated to be a complex synergistic effect of three major flavonol aglycones and their mono glucosides, as the anti-inflammatory effect was better than that of other fractions. In addition, three mono-glycoside flavonols such as kaempferol-3-glucoside, quercetin-3-glucoside, and isorhamnetin-3-glucoside, were demonstrated to be PPAR-α activators by molecular docking simulation. Together, these results suggest that the flavonol composition of *E. sativa* can be used as a therapeutic agent for maintaining healthy skin by alleviating inflammatory skin diseases such as atopic dermatitis.

## Figures and Tables

**Table 1:** DPPH radical scavenging activity, total polyphenol contents, and total flavonoid contents of *E. sativa* fractions (** *p* < 0.01, *** *p* < 0.001, compared with ES).

Sample	SC_50_ (µg/mL)	GAE (mg/g)	QE (mg/g)
ES	610.40	32.22 ± 2.70	38.46 ± 15.55
EEA	229.38 ***	104.99 ± 5.88 ***	74.24 ± 1.67 **
EBuOH	494.93 **	88.42 ± 2.17 ***	53.41 ± 0.33
ECHCl_3_	650.19	44.35 ± 3.55	39.30 ± 8.29
EDW	1993.50	16.58 ± 0.89	27.43 ± 8.18
EHEX	2078.39	8.52 ± 1.00	44.73 ± 14.31
L-ascorbic acid	23.86 ***	-	-

**Table 2:** The inhibition of NO production of flavonols.

Sample	IC_50_ (μM)
Quercetin-3-glucoside	1.81
Kaempferol-3-glucoside	58.33
Isorhamnetin-3-glucoside	-
Quercetin	72.27
Kaempferol	89.38
Isorhamnetin	442.02

**Table 3:** Identification of flavonols of each fraction by LC/MS data based on MS2 fragmentation pattern.

No.	T_R_ (min)	Formula	Ion	Experimental *m*/*z*	Calculated *m*/*z*	Fragment	Identification
1	6.2	C_27_H_30_O_17_	[M^-^H]^-^	625.25	625.14	463, 301	Quercetin 3,4′-diglucoside
2	6.9	C_27_H_30_O_16_	[M^-^H]^-^	609.25	609.15	477, 315	Kaempferol 3,4′-diglucoside ^*^
3	7.9	C_28_H_32_O_17_	[M^-^H]^-^	639.25	639.16	447, 285	Isorhamnetin 3,4′-diglucoside ^*^
4	11.4	C_21_H_20_O_12_	[M^-^H]^-^	463.17	463.09	301	Quercetin-3-glucoside
5	14.6	C_21_H_20_O_11_	[M^-^H]^-^	447.17	447.09	284, 255, 327	Kaempferol 3-glucoside
6	15.4	C_22_H_22_O_12_	[M^-^H]^-^	477.17	477.10	314, 357, 285	Isorhamnetin 3-glucoside
7	25.1	C_15_H_10_O_7_	[M^-^H]^-^	301.08	301.03	179, 151, 273	Quercetin
8	32.6	C_15_H_10_O_6_	[M^-^H]^-^	285.08	285.03	151, 257, 229, 185	Kaempferol
9	33.9	C_16_H_12_O_7_	[M^-^H]^-^	315.07	315.05	300	Isorhamnetin

* The identification was checked by the previous study on *E. sativa* referred to reference [9].

**Table 4:** Flavonols content of each fraction of *E. sativa* by HPLC-UV (% based on each fraction, N.D.: not detected).

Flavonols Detected	Crude Extract (by 70%EtOH)	Hexane Fraction	CHCl_3_ Fraction	Ethyl Acetate Fraction	Butanol Fraction
Quercetin 3,4′-diglucoside	0.062 ± 0.002	0.049 ± 0.004	N.D.	0.25 ± 0.02	1.05 ± 0.009
Kaempferol 3,4′-diglucoside	0.22 ± 0.006	0.19 ± 0.002	0.010 ± 0.0009	1.26 ± 0.05	5.70 ± 0.05
Isorhamnetin 3,4′-diglucoside	0.048 ± 0.003	0.020 ± 0.002	N.D.	0.12 ± 0.004	0.37 ± 0.02
Quercetin 3-glucoside	0.0055 ± 0.0003	0.0045 ± 0.0003	0.0021 ± 0.0002	2.27 ± 0.07	0.20 ± 0.003
Kaempferol 3-glucoside	0.018 ± 0.0004	0.016 ± 0.0007	N.D.	7.37 ± 0.3	0.31 ± 0.004
Isorhamnetin 3-glucoside	0.0072 ± 0.0003	0.0031 ± 0.0001	N.D.	1.44 ± 0.08	0.099 ± 0.001
Quercetin	N.D.	N.D.	0.0016 ± 0.00007	0.093 ± 0.008	0.0014 ± 0.00009
Kaempferol	0.00071 ± 0.00004	N.D.	0.0042 ± 0.0002	0.32 ± 0.02	N.D.
Isorhamnetin	N.D.	N.D.	0.010 ± 0.0005	0.13 ± 0.006	N.D.
Sum	0.36	0.28	0.03	13.2	7.7

## Data Availability

Data is contained within the article.
